# Eosinophilic Pleural Effusion in Gnathostomiasis

**DOI:** 10.3201/eid1009.030671

**Published:** 2004-09

**Authors:** Philippe Parola, Gerard Bordmann, Philippe Brouqui, Jean Delmont

**Affiliations:** * Hopital Nord, Marseille, France;; † Swiss Tropical Institute, Basel, Switzerland

**Keywords:** letter, Gnathostomiasis, imported disease, travelers, Vietnam

**To the Editor:** Moore et al. reported a case series of patients infected with imported gnathostomiasis who had typical intermittent, migratory skin manifestations or peripheral blood eosinophilia or both, as well as undiagnosed eosinophilia with nonspecific symptoms (*1*). We would like to add some comments based on a recent patient treated in Marseille, France.

In April 2003, a 66-year-old man living in Marseille indicated a history of fever for 8 days. He had returned from Vietnam 1 month earlier, where he had stayed for 4 weeks in the Ho Chi Minh City area and 2 days in the Mekong Delta area. He was well during his trip, and reported no arthropod bites except from mosquitoes. He had no direct skin contact with river water. Dietary intake included local dishes with rice, fish, pork, shrimp, and chicken. His symptoms started 3 weeks after he returned to Marseille and included fever (temperature 38°C), asthenia, chills (1 day), moderate dyspnea during exercise, transient bilateral pain of the testes, and an episode of hemospermia. He was referred by his family doctor to the Infectious and Tropical Diseases Unit, North Hospital.

On admission, the patient's temperature was 38°C. Physical examination of the patient, including the testes, was normal except for a systolic heart murmur (preexisting and known to the patient), and clinical signs of left pleural effusion. The effusion was subsequently confirmed by chest x-ray, which also showed a discrete diffuse bilateral lung infiltrate. Results of routine laboratory tests conducted on blood samples were normal except for an elevated eosinophil count of 5.2 10^9^/L. Blood smears for plasmodia and microfilaremia were negative. Urologic examination, including echography and prostate-specific antigen, showed no abnormalities except a prostatic adenoma (preexisting and known to the patient). No eggs or parasites were detected by microscopic examination in stools or in urine, although both sedimented and centrifuged urine specimens were studied and filtration techniques were used. After transthoracic aspiration of 100 mL of pleural effusion, cytologic examination showed an eosinophil count of 5,800/L without parasites. Bacterial culture, including mycobacteria, was negative. On day 4, the patient was afebrile and was discharged. On admission, results of a first set of examinations involving reactivities to schistosomiasis, paragonimiasis, strongyloidiasis, cysticercosis, trichinosis, gnathostomiasis, filariasis, and toxocariasis were negative.

One month later, the eosinophil count of our patient had decreased to 1.8 x 10^9^/L. He was afebrile, and his only complaint was asthenia. A new set of serologic examinations was conducted. The Western blot assay for gnathostomiasis conducted at the Swiss Tropical Institute (Socinstrasse 57, CH-4002, Basel, Switzerland) was positive, showing immunoglobulin G reactivity to four specific bands including the 24-kDa band, considered pathognomonic for the diagnosis of *Gnathostoma* infection (*1*). The seroconversion confirmed the diagnosis of gnathostomiasis. All other serologic tests remained negative, except an increase of antibodies against *Acanthocheilonema vitae* used as antigen for unspecific serologic screening for filariasis (Laboratoire Marcel Merieux, Lyon, France). After a 21-day course of albendazole and a single dose of ivermectin, the eosinophil count of our patient decreased to 0.8 x 10^9^/L.

Three aspects of gnathostomiasis as an emerging imported disease can complement the findings of Moore et al. (*1*). First, the clinical findings in our case are very unusual. Hemospermia is often benign with predominant causes including prostatic and seminal vesicle disease. Infections, including mainly schistosomiasis and tuberculosis, have been associated with these symptoms (*2*). Although our patient had a prostatic adenoma, this is the first time that hemospermia has been associated with gnathostomiasis. Because of the anxiety hemospermia caused, this symptom was the main reason that our patient consulted our center. Secondly, eosinophilic pleural effusion is also unusual in gnathosthosmiasis. Although reported as a potential cause in reference books (*3*), a MEDLINE search (key words: gnathostomiasis and eosinophilia and pleural effusion or pleuritis or lung) disclosed only two references to pleural effusion as the main symptom of gnathostomiasis (*4**,**5*). The eosinophilic pleural and pulmonary response may be elicited by the larvae of helminths carried hematogenously into lungs and pleura in an aberrant fashion (*3*). The last point we stress is that, as shown by Moore et al. (*1*), patients returning from disease-endemic areas, mainly Southeast Asia and Central and South America, should be tested systematically for gnathostomiasis. Although some patients show a typical cutaneous form of gnathostomiasis associated with eosinophilia (*6**,**7*), most atypical forms are probably underdiagnosed, and severe neurologic involvement may occur if treatment is not given (*1*). However, until recently specific serologic tests for gnathostomiasis were available only in Asia, mainly in Thailand and Japan. Some laboratories in Europe currently provide testing for gnathostomiasis, which would be a valuable aid in evaluating patients returning from the tropics.
